# Structure Strengthening Phenomena of Gluten Matrices under Different Stress Types

**DOI:** 10.3390/polym15234491

**Published:** 2023-11-22

**Authors:** Leonhard Maria Vidal, Thekla Alpers, Thomas Becker

**Affiliations:** Research Group Cereal Technology and Process Engineering, Institute of Brewing and Beverage Technology, Technical University of Munich, 85354 Freising, Germany; thekla.alpers@tum.de (T.A.); tb@tum.de (T.B.)

**Keywords:** strain hardening, dough, wheat flour, baking performance, rheology, dough, rheology, strain hardening, gluten network, wheat flour

## Abstract

To predict the achievable product volume with respect to the gas retention capacity of the gluten matrix in wheat flour doughs, strain hardening evaluation is crucial. But assessing these structure hardening phenomena in wheat flour dough systems is still a challenging task. In this work, a simple shear method applied to kneaded dough samples was tested and compared to biaxial extension tests performed with a lubricated squeezing flow method. The comparability of shear-induced structure hardening with biaxial extension tests was shown. Structure hardening and breakdown after overload were visualized using shear flow and a comparison of the obtained shear flow over Hencky strain curve peaks. To predict the behavior of the analyzed flours according to their composition, a correlation analysis of the flour and dough properties was performed. The influence of the HMW glutenin subunits on the sensitivity of the dough matrix according to the applied shear speed (0.1 and 1.0 mm/s) could be shown with a correlation coefficient of 0.94. The LMW glutenin subunits, on the other hand, showed a high correlation coefficient of 0.89 with the achievable network connectivity parameter z [-] gained from frequency sweep testing.

## 1. Introduction

Strain hardening is a complex phenomenon that occurs in wheat flour dough as it is subjected to mechanical forces, such as mixing or kneading. The physical and chemical changes that take place during this process are critical to the development of the dough’s structure, as well as its ability to retain gas and water. Mixing wheat flour or gluten–starch blends with water leads to a unique viscoelastic matter. The evolving dough owes these viscoelastic properties to its crosslinked protein network and enables a variety of food applications [1]. In the beginning of the dough kneading procedure, the mechanical energy input together with the hydration of the flour/gluten particles causes the crosslinking of glutenins and gliadins into a continuous protein network with sizes between 150 kDa and 1500 kDa [2]. One key reaction is the oxidation of sulfhydryl groups on the gluten proteins, which leads to the formation of disulfide bonds between adjacent protein strands. These bonds contribute to the dough’s strength and elasticity, as they support the protein network interconnections. This network is known as the continuous gluten phase [3,4]. Both during kneading and further processing of the dough, strain hardening can be observed when the dough is subjected to a constant load. Strain hardening occurs if a sample is subjected to an increasing strain at a constant strain rate. This phenomenon is defined as when the stress required to deform a material increases to more than the resulting strain at constant deformation [5]. Since it was found that strain hardening in dough is strongly affected by the gluten network and responsible for the gas retention capacity, extensional or biaxial compression tests were implemented to evaluate these effects for dough systems [6,7,8,9]. Regarding the time- and load-dependent behavior of wheat flour doughs, an increasing resistance to shear stress can also be observed. If we consider the structural changes in the dough under a given load, several changes could be responsible for strain hardening: Firstly, the entangled polymer strands are orientated in load direction and accumulate closer together [10]. This results in more hydrogen and van der Waals-type bonds between the strands. Secondly, the agglomerated gliadins break down to primary particles, which become entrapped in the crosslinked glutenin strands. Interaction with the strands creates a more interconnected network. Additionally, the starch–starch and starch–gluten interactions are slightly deformed and store some of the applied load [11]. Until the maximum achievable particle deformation is reached, the strain can increase, and with higher loads, the resistance of the dough also increases. Meerts et al. stated that as the load increases, the short-range gluten–starch and starch–starch interactions in the dough begin to break down to the point where eventually only the long-range gluten–gluten interactions remain to provide structural integrity to the material [12,13]. Especially when considering the reversible entropy–elastic behavior of entangled polymers, wheat flour dough can be compared with other polymeric networks when a load is applied, and strain hardening occurs [14]. The breaking, or the shift, of molecular bonds under load is a widespread polymeric behavior. This process results in an increase in the material’s shear modulus and tensile strength, as well as a decrease in its extensibility at break. Regarding this assumption of comparability, a definition of a strain hardening index (SHI) similar to an equation for polypropylene blends was defined for extensional stress and was experimentally validated [15]:

(1) $$SHI=\frac{\eta_{e}^{+}(\varepsilon_{max})}{\eta_{e0}^{+}(\varepsilon_{max})}$$

where the maximum strain is *ε_max_* and $\eta_{e}^{+}(\varepsilon_{max})$ represents the actual value of the transient extensional viscosity at the maximum strain, and $\eta_{e0}^{+}(\varepsilon_{max})$ is the value of the extrapolated LVE at maximum strain. This index reaches a maximum for kneaded doughs close to the optimum development stage [13]. At the optimum development stage, the network best withstands the applied deformations of the kneader geometry [16]. Since the kneading process is not only based on extension, shear forces also play a decisive role in network development during dough kneading [17]. To bridge the gap between strain hardening evaluation and shear-induced matrix hardening, shear flow and stress–strain curve evaluation were investigated for their predictive quality in the hardening and baking performance of wheat flours. In addition to the evaluation of the predictive capabilities of the calculated strain hardening indices, flour composition in the form of Osborne fractions and dough properties such as dough development time or the microscopically analyzed total number of protein junctions in the matrix are considered.

The aim of this study was to evaluate the value of a shear-only structure hardening test to describe gluten-phase strengthening under different load types. Also, a principal component analysis of flour, dough and bread properties was conducted to determine the accuracy of shear testing and classical analysis of the network attributes. The evaluated shear-stress-induced structure strengthening testing method could be an additional tool to evaluate flour and dough properties in line with a conventional rheometer. With respect to the previous work of Vidal et al. [16,18,19], microstructural evolution and flour properties were linked with dough processing behavior. Taking a broad overview of baking-performance-determining attributes into account, correlations between these flour and dough properties were revealed. The evaluated shear-stress-induced structure strengthening testing method provides a precise tool with which to evaluate flour and dough properties in line with a conventional rheometer. 

## 2. Materials and Methods

For all experiments, commercial wheat flours type 0 and type 00 provided by Rieper AG, Vintl, Italy were used. The composition (see Table 1) was previously analyzed [16,18,19].

For the shear rheological measurements of dough in the MCR502 (Anton Paar, Ostfildern, Germany), a specific amount of each flour (corrected to 14% moisture) and demineralized water were kneaded at 63 rpm using a z kneader, DoughLAB (Perten Instruments AB, Hägersten, Sweden) equipped with a 50 g mixing bowl [18]. For the lubricated squeezing flow (LSF) measurements on a TA.XT.Plus with a 50 kg load cell (Stable Microsystems, Godalming, UK), a 300 g mixing bowl with adapted flour weight and the maximum water dosage was used. In the MCR502, a 4 g sample piece was compressed to a 2 mm gap size according to the lower and upper rheometer geometry (serrated plates, Ø 25 mm), the dough overhang was removed with a scalpel and the edges were covered with paraffin oil to prevent drying. The measuring environment during the shear rheological experiments was set to 30 °C and 80% RH in the MCR502. For the LSF measurements, the temperature was set to 30 °C with no humidity control. 

### 2.1. Strain Hardening Index from Biaxial Extension 

In order to calculate the biaxial strain *ε_b_* (1) and biaxial strain rate $\dot{\varepsilon }$*_b_* (2) according to Chatraei et al. [20], the dough samples were placed between two plates 45 mm in diameter in a texture analyzer (TA.XT.Plus, Stable Microsystems, Godalming, UK) equipped with a 50 kg load cell.

(2) $$\varepsilon_{b}=-\frac{1}{2}\cdot \ln\left(\frac{h\left(t\right)}{h_{0}}\right)$$

(3) $${\dot{\varepsilon }}_{b}=\frac{v}{2h(t)}$$

In (1) and (2), *h*_0_ and h_t_ are the initial sample thickness and the thickness at time *t* with v as the compression speed. The samples, having the same diameter as the two plates, were compressed from their initial height of 20 mm to 2 mm at displacement speeds of 0.1, 1, 2, 5 and 10 mm/s. To obtain the strain hardening index as proposed by [21,22,23], the force *F*, determined from the measurements, can be converted into the stress *σ* using the area given by the specimen geometry *A* (as $\pi *r^{2}$, where r is the diameter of the sample) and the following equation.

(4) $$\sigma =\frac{F}{A}$$

For the given deformations of 0.3, 0.4, 0.5, 0.6, 0.7, 0.8, 0.9 and 1.0, the stress values were extracted for each measurement at the five displacement speeds. This was achieved by plotting these stress values against the biaxial strain rate on a double logarithmic scale. The deformation data were then fitted with a linear model. Using this regression model, stress values were calculated for specific values of ${\dot{\varepsilon }}_{b}$ (0.1, 1.0) and plotted against deformation on a logarithmic scale. The slope of the linear fit of the plotted data is defined as the strain hardening index (SHI) expressed by Equation (4) [21,23]:

(5) $$SHI={\left(\frac{\delta \ln(\sigma )}{\delta \varepsilon_{b}}\right)}_{{\dot{\varepsilon }}_{b}=const.}$$

### 2.2. Strain Hardening from Shear 

By fitting the stress–strain curve obtained from shear testing the dough samples in the MCR502 with shear speeds of 0.1 and 1.0 mm/s, the Equation (5) was utilized to calculate the strain hardening exponent [24]:

(6) σ=K∗exp(n∗ε)

where n stands for the strain hardening exponent and *K* for the strength coefficient. Both can be calculated by fitting the empirical exponential equation, in which *σ* is the measured stress and *ε* the calculated Hencky strain. The Hencky strain from shear can be calculated with Equation (6), taking into account the shear rate $\dot{\gamma }$, which was set in additional experiments to fixed values of 0.1 and 1.0 s^−1^, and test time t [25]:

(7) $$\varepsilon_{h}=\frac{1}{2}\;\ln\left(1+\frac{{\dot{\gamma }}^{2}t^{2}}{2}+\dot{\gamma }t\;\sqrt{1+\frac{1}{4}\;{\dot{\gamma }}^{2}t^{2}}\right)$$

Following Tanner et al. [26], one can find the viscometrical functions for shearing with the shear rate $\dot{\gamma }$ beginning at *t* = 0, with the same form of shear rate and strain dependence as seen in elongation [27]:

(8) $$\tau =\frac{G\left(1\right)}{1-p}\;{\dot{\gamma }}^{p}{\left(\gamma \right)}^{1-p}$$

where $\gamma =\dot{\gamma }*t$ and *p* is a constant. From oscillation measurements, it is also known that *G*(*t*) is the relaxation function and $\dot{\gamma }$ is the shear rate. It is known that the form of *G*(*t*) therefore corresponds to (8) with the constant *p* [28]:

(9) $$G\left(t\right)=G\left(1\right)*\;t^{-p}$$

By calculating the shear flow (9) by fitting the stress curve with the calculated constant *p* and the strain rate, the determination of structure breakdown at the peak can be visualized:

(10) $$Shear\;Flow=\frac{\tau }{{\dot{\gamma }}^{p}}$$

### 2.3. Statistical Analysis

Statistical evaluation was performed with OriginPro2022b (OriginLab, Northampton, MA, USA) using Pearson correlations and one-way and two-way analysis of variance (ANOVA) with Tukey’s post hoc test at a level of significance of *p* < 0.05.

## 3. Results and Discussion

Strain hardening indices can be calculated using different methods. Different methods may lead to different results, as the prerequisites are not always fulfilled, and difficulties in the accurate determination of strain and stress, variable strain rates and the use of different equations might arise [5,24]. As the strain hardening index is dependent on the method used, we focused on the comparison of biaxial extension and shear flow for the Results section. The results were then analyzed according to their correlations with previously analyzed flour and dough properties. 

### 3.1. Biaxial Extension

**Figure 1:**
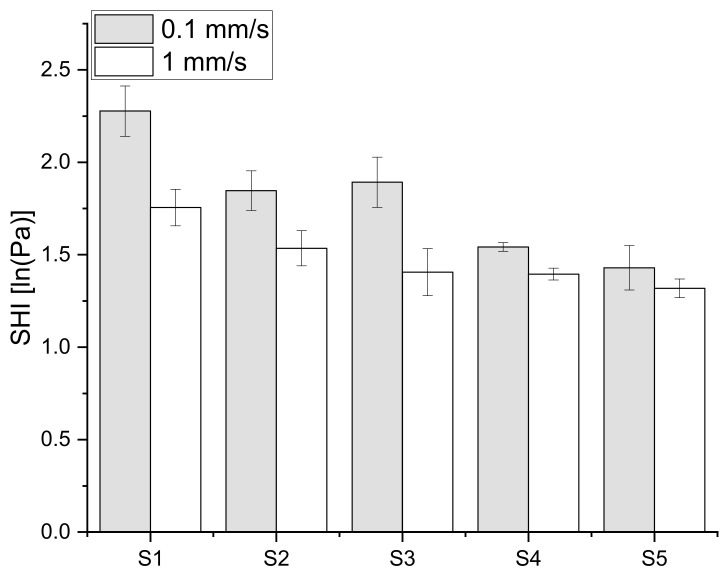
Strain hardening indices (SHIs) for five kneaded dough samples. The SHI is determined via compression tests and the resulting biaxial extension. Results are shown for the respective 0.1 and 1 mm/s elongation rate ${\dot{\varepsilon }}_{b}$. (n = 3).

Under constant load, wheat flour dough exhibits strain hardening as well as strain rate hardening. Previous works underlined the influence of the strain rate, as it affects the behavior of the entangled coupling of large glutenin molecules at the molecular level [24,29]. The SHI is therefore dependent on the strain rate, which can be seen in Figure 1. The higher extension speed revealed that for faster deformation, the lower-protein-containing flours showed comparable hardening. No significant differences were found between S3 and S5 for the extension speed of 1 mm/s. For the slower extension speed, the influence of the protein content was stronger. Less strong and less interconnected protein strands (in the lower-protein-containing flours) were not able to align in the same manner as the higher-protein samples in the direction of the applied stress to form new bonds or to orientate along the direction of deformation. With respect to the protein contents in this work and considering the trend of a lower ratio of higher speed to lower speed Shi, the lower-protein-containing flours may lead to a reverse ratio between SHI for slow and higher biaxial extensions [30]. 

### 3.2. Shear Experiments 

#### 3.2.1. Dependency of the Strain Hardening Exponent on Deformation Speed 

**Figure 2:**
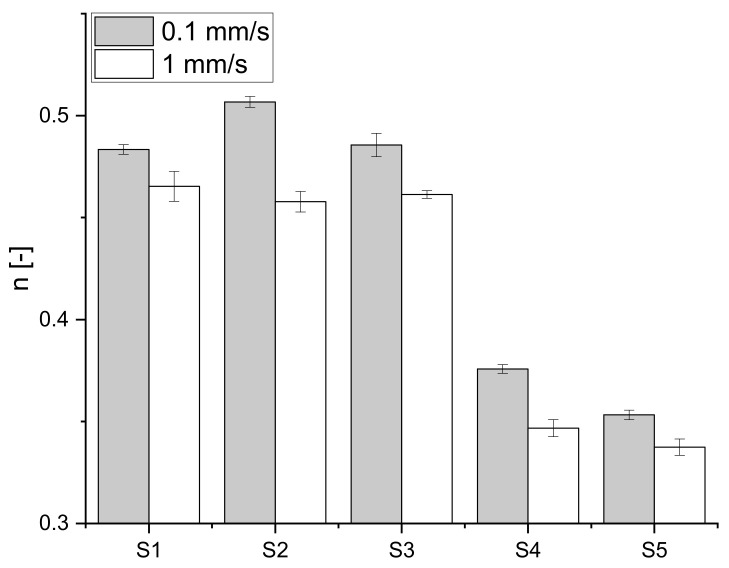
Strain hardening exponent, n, obtained via power-law-fitting the results of the stress–strain curves for five flour samples sheared in a rheometer at deformation speeds of 0.1 and 1 mm/s. (n = 3).

As shown for biaxial extension in Figure 1, as well as for the shear load experiments in Figure 2, the lower deformation speed showed higher stiffening. This may be caused by the internal relaxation processes, which allow the material to adapt to the applied load and rearrange the protein strands during their deformation in the shear direction [31]. At a higher speed, the structure may become damaged even before the alignment can take place properly. For the shear load experiments, the higher-protein-containing flours (S1–S3) showed comparable behavior for the faster deformation rate. Therefore, the higher protein content enabled the dough matrix to withstand deformation better and led to a stronger network. The reason for this behavior may be that stronger flours and the resulting stronger doughs tend to contain more HMWs [19]. Therefore, the strength of the secondary forces is higher due to the larger chains, and these doughs need higher shearing to stretch the gluten molecules to the maximum and cause bond scission or the opening of entanglements [32]. 

#### 3.2.2. Increase in the Shear Flow Depending on Hencky Strain and Flour Composition 

**Figure 3:**
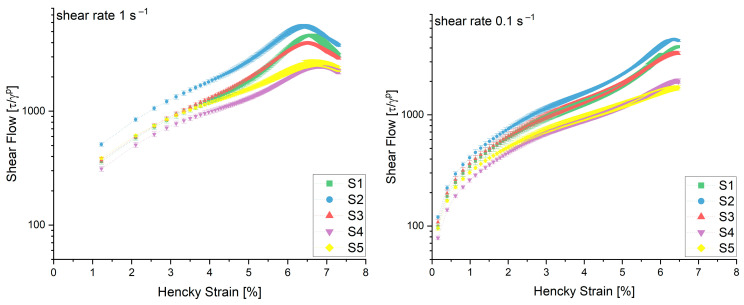
Calculated shear flow over the Hencky strain for five flour samples at a shear rate of 1.0 and 0.1 s^−1^. (n = 3).

In Figure 3, the shear flow curves calculated from the simple shear load test showed a dependence on the flour composition as well as the applied shear rate. The peak of the curve, determining the structural breakdown of the sample, shifted to lower Hencky strains but higher shear flow values with decreasing protein content. This can be explained by the fact that with increasing strain, the short-range gluten–starch and starch–starch interactions in dough start to break down. This breakdown may happen to such an extent that only the longer-range gluten–gluten interactions remain to provide structural integrity [12,13]. On the other hand, for higher-protein-containing flours, the difference in the possibility of withstanding these deformations and the adaption to the applied load through structure strengthening is not as pronounced as for weaker flours. As mentioned before, the secondary forces interconnecting the protein strands may be more pronounced due to the higher HMW content and therefore larger chains. Comparing the two methods analyzed in this work, the shear method was able to identify the gas retention capability of the flour samples in the same manner as the biaxial extension. For the lower shear rate of 0.1 s^−1^, the shear flow evaluation method was even more precise in connecting the achievable product volume with the protein content (see Table 2) with a correlation coefficient of 0.84. 

### 3.3. Correlation Analysis of Flour and Dough Parameters 

To take all the analyzed parameters of the flours used in this work along with the previous works of Vidal et al. into account, a correlation analysis was carried out. Table 2 lists the correlation coefficients. The red-marked coefficients correlated with a level of significance of *p* < 0.05. The results show that the dough development time (DDT) had a strong correlation of 0.90 with the total number of junctions (TNoJ) as determined via microscopic analysis. This can be explained by the fact that more contained protein, and in the case of the analyzed flours, also more high-molecular-weight subunits (HMWs) can lead to more intermolecular bonds between protein strands [33,34]. As a result, a high correlation of 0.94 between the HMW and the TNoJ could be observed. The TNoJ also had a strong correlation of 0.88 with the maximum peak of the shear flow curve occurring at the 0.1 mm/s shear speed. This peak represents the structure breakdown point of the matrix and is also strongly correlated with the HMWs for both shear speeds. In addition to the peak of shear flow, the TNoJ correlates strongly (0.90) with the SHI calculated for the slower biaxial extension speed of 0.1 mm/s. For the faster shear speed of 1.0 mm/s, the correlation with the TNoJ was even higher at 0.98. With respect to this, one can clearly see that the strongly crosslinked proteins play a pivotal role during structure strengthening as well as during network breakdown. On the other hand, the low-molecular-weight subunits (LMWs) showed a high correlation of 0.89 with the achievable network connectivity z [-] at the optimum development stage of the network evolution of the kneaded dough samples. Therefore, LMWs may be a crucial binding partner for HMWs within the network in which the matrix best withstands the deformation applied by the kneader geometry. Until the optimum development stage, HMWs do not play a significant role due to their lower molecular mobility, which is caused by bigger and more extensive intermolecular disulfide bonds [33]. These bonds reduce the mobility of the HMW, and therefore, the influence on network connectivity, z, is mainly based on the LMW at this stage of dough development. The Albumine and Globuline content showed strong negative correlations with the calculated exponent n at speeds of 0.1 and 1.0 mm/s. This can be explained by the viscosifying nature of these subunits due to their solubility in water and their ability to form polymeric connections with other proteins, entrapping water in the matrix [35,36]. The Omega b subunits showed strong positive correlations with the exponent n as well as the HMW at 1 mm/s. The influence of the HMW on the exponent n may be explained by the strong disulfide bindings of the gluten network and could be caused by the low molecular mobility of these molecules, as mentioned before. In a direct comparison of the biaxial extension and shear-dependent structure hardening phenomena, the SHI and the exponent n showed, at both deformation speeds, high correlations between 0.81 and 0.95. With these findings, the comparability of the shear-induced structure hardening evaluation with other testing methods was demonstrated, and the shear setup was determined to be even more sensitive to the protein contents of the flour samples. Compared to the volume increase in classical baking trials, the shear method using the slower shear rate of 0.1 s^−1^ showed strong correlations of 0.84 for the calculated shear flow peak and 0.89 for the strain hardening exponent n. The biaxial extension method was not able to determine the baking performance of the flour samples as precisely as the shear method and showed no such strong correlations.

## 4. Conclusions

By applying constant shear load on wheat flour dough samples with a rheometer, the strain hardening induced by shear load was directly comparable to biaxial extension. Based on fundamental rheology, structure hardening could be observed in the same manner as under the biaxial extension applied. Therefore, the shear flow calculation and the power law fitting of shear-induced stress–strain curves represent a useful method for determining the quality parameters of wheat flours. The controlled energy input together with the small sample size and limited sample handling provides a good alternative to lubricated squeezing flow methods. For the shear flow calculations, the dependency of the protein content (especially HMWs), with a negative correlation of up to −0.95 with the albumin/globuline content of the peak of the curve achieved, and the suitability of the shear rheological measurement to obtain flour/dough strength under load could be demonstrated. With respect to previous works, the findings of this work show strong correlations between flour attributes such as dough development time (DDT) and total number of junctions (TNoJ). The developed dough matrix demonstrated the strongest correlation of structure strengthening under shear with the total number of junctions. For the biaxial extension, the same magnitude of correlation could be observed for TNoJ as well as for the DDT at a lower extension rate of 0.1 mm/s. In a direct comparison of simple shear (calculated as shear flow peak) to biaxial compression SHI, the 0.1 mm/s as well as the 1.0 mm/s shear and extension rates showed strong (0.87 and 0.82) and medium (0.78 and 0.67) correlations with the same deformation speeds. This underlines the applicability of the setup to further investigate dough strengthening under shear load. Since results were obtained only for a small batch of commercial wheat flours, the applicability of the shear-induced structure strengthening testing method must be further investigated, especially for lower-protein-containing flours. Since the total amount of protein in commercial flours may be trending downwards due to limitations in fertilization or precipitation, a precise and simple-to-use technique to predict the gas retention capacity of wheat flour doughs, and therefore the baking characteristics of the matrix, is needed to maintain high product quality and consumer acceptance of baked goods.

## Figures and Tables

**Table 1:** Flour samples and their protein content [18,19].

Flour	S1	S2	S3	S4	S5
Protein (g/100g)	14.86 ± 0.06	14.29 ± 0.03	14.22 ± 0.01	11.79 ± 0.03	11.77 ± 0.05
ω5-Gliadin	3.9	3.6	3.9	3.9	3.3
ω1,2-Gliadin	3.8	3.8	4	3.9	3.5
α-Gliadin	31.2	33	32.2	32.3	29.6
γ-Gliadin	20.8	20.4	20.6	22.1	21.4
HMW-GS	8.6	8.6	8.1	5.9	6.6
LMW-GS	19.9	19.3	18.7	17.5	19

**Table 2:** Correlation coefficient of linear correlations between dough process parameters and Osborne fractions of the analyzed flours. Significant correlations are marked in red color (*p* < 0.05).

	DDT	Network Connectivity	TNoJ	Peak Shear Flow 0.1 1/s	Peak Shear Flow 1 1/s	K 0.1 mm/s	n 0.1 mm/s	K 1 mm/s	n 1 mm/s	SHI 0.1 mm/s	SHI 1 mm/s	Gliadine	Glutenine	Albumine /Globuline	Omega 5	Omega 1,2	Alpha	Gamma	Omega b	HMW	LMW	Volume Increase in Baking Trials
DDT	1																					
Network Connectivity	0.19	1																				
TNoJ	0.90	0.24	1																			
Peak Shear Flow 0.1 1/s	0.63	0.35	0.88	1																		
Peak Shear Flow 1 1/s	0.54	0.48	0.82	0.98	1																	
K 0.1 mm/s	−0.43	0.05	−0.71	−0.88	−0.82	1																
n 0.1 mm/s	0.77	0.18	0.85	0.86	0.79	−0.84	1															
K 1 mm/s	−0.94	−0.07	−0.94	−0.78	−0.68	0.70	−0.92	1														
n 1 mm/s	0.84	0.30	0.98	0.95	0.90	−0.80	0.91	−0.92	1													
SHI 0.1 mm/s	0.90	0.02	0.90	0.78	0.67	−0.75	0.95	−0.99	0.90	1												
SHI 1 mm/s	0.51	0.12	0.70	0.87	0.82	−0.94	0.94	−0.75	0.81	0.81	1											
Gliadine	−0.06	−0.77	0.09	0.21	0.09	−0.63	0.32	−0.23	0.14	0.32	0.49	1										
Glutenine	0.76	0.69	0.87	0.86	0.87	−0.53	0.75	−0.74	0.89	0.69	0.62	−0.30	1									
Albumine/Globuline	−0.67	−0.09	−0.88	−0.95	−0.89	0.96	−0.93	0.86	−0.94	−0.88	−0.94	−0.45	−0.72	1								
Omega 5	0.51	−0.60	0.49	0.39	0.23	−0.67	0.68	−0.70	0.49	0.76	0.65	0.80	0.08	−0.65	1							
Omega 1,2	0.48	−0.68	0.54	0.39	0.23	−0.61	0.48	−0.62	0.48	0.65	0.44	0.76	0.05	−0.61	0.87	1						
Alpha	0.10	−0.46	0.40	0.58	0.51	−0.87	0.51	−0.40	0.46	0.47	0.69	0.88	0.09	−0.72	0.69	0.75	1					
Gamma	−0.65	−0.50	−0.87	−0.88	−0.89	0.59	−0.60	0.65	−0.87	−0.59	−0.53	0.15	−0.90	0.74	−0.06	−0.25	−0.30	1				
Omega b	0.96	0.25	0.97	0.82	0.74	−0.64	0.89	−0.98	0.95	0.95	0.70	0.05	0.85	−0.84	0.54	0.50	0.29	−0.77	1			
HMW	0.78	0.52	0.94	0.94	0.94	−0.69	0.81	−0.81	0.97	0.78	0.72	−0.08	0.97	−0.85	0.24	0.27	0.31	−0.94	0.90	1		
LMW	0.61	0.89	0.64	0.63	0.69	−0.23	0.54	−0.52	0.67	0.46	0.39	−0.60	0.93	−0.44	−0.22	−0.30	−0.26	−0.74	0.66	0.81	1	
Volume Increase in Baking Trials	0.49	−0.13	0.78	0.84	0.77	−0.88	0.89	−0.66	0.65	0.66	0.70	0.53	0.52	−0.88	0.55	0.74	0.83	−0.75	0.65	0.71	0.18	1

## Data Availability

The data presented in this study are available on request from the corresponding author.
